# Faster visual reaction times in elite athletes are not linked to better gaze stability

**DOI:** 10.1038/s41598-020-69975-z

**Published:** 2020-08-06

**Authors:** Brendan T. Barrett, Alice G. Cruickshank, Jonathan C. Flavell, Simon J. Bennett, John G. Buckley, Julie M. Harris, Andrew J. Scally

**Affiliations:** 1 School of Optometry and Vision Science, University of Bradford, Bradford, BD7 1DP West Yorkshire UK; 2 Department of Psychology, University of York, York, YO10 5DD North Yorkshire UK; 3 School of Sport and Exercise Science, Liverpool John Moores University, Liverpool, L3 3AF UK; 4 Department of Biomedical and Electronics Engineering, University of Bradford, Bradford, BD7 1DP West Yorkshire UK; 5 School of Psychology and Neuroscience, University of St Andrews, St Andrews, KY16 9JP UK; 6 School of Clinical Therapies, University College Cork, College Road, Cork, T12 K8AF Republic of Ireland

**Keywords:** Human behaviour, Oculomotor system, Sensory processing, Visual system

## Abstract

The issue of whether visually-mediated, simple reaction time (VRT) is faster in elite athletes is contentious. Here, we examined if and how VRT is affected by gaze stability in groups of international cricketers (16 females, 28 males), professional rugby-league players (21 males), and non-sporting controls (20 females, 30 males). VRT was recorded via a button-press response to the sudden appearance of a stimulus (circular target—diameter 0.8°), that was presented centrally, or 7.5° to the left or right of fixation. The incidence and timing of saccades and blinks occurring from 450 ms before stimulus onset to 225 ms after onset were measured to quantify gaze stability. Our results show that (1) cricketers have faster VRT than controls; (2) blinks and, in particular, saccades are associated with slower VRT regardless of the level of sporting ability; (3) elite female cricketers had steadier gaze (fewer saccades and blinks) compared to female controls; (4) when we accounted for the presence of blinks and saccades, our group comparisons of VRT were virtually unchanged. The stability of gaze is not a factor that explains the difference between elite and control groups in VRT. Thus we conclude that better gaze stability cannot explain faster VRT in elite sports players.

## Introduction

The perceptual factors underlying the highest levels of elite sporting performance are attracting considerable research attention. There is a large and growing volume of work that suggests perceptual-cognitive expertise is a crucial component in the elite advantage, reflected by their knowledge of precisely where and when to look to gather key information^1–11^. This information enables elite players to better anticipate upcoming events and, consequently, plan and execute optimal motor responses (e.g. hitting a ball or passing to a teammate).

Another factor which continues to receive much attention in elite sport is reaction time. In its simplest form, visually-mediated reaction time can be defined as the time taken to respond (typically via a button press) to the sudden appearance or change of a visual stimulus. This is referred to as ‘simple’ reaction time, to distinguish it from ‘choice’ reaction time. Unlike simple reaction time, choice reaction time requires a choice to be made regarding how to respond (e.g., by pressing one of four keys with a specific digit depending on which of several stimuli was presented). Since simple reaction times involve lower processing demands, they are faster than choice reaction time. The focus of the present study is to measure simple reaction time to a visual stimulus (hereafter, ‘VRT’). There are many examples of time-limited sporting scenarios in which a rapid motor response appears to be coupled to the sudden appearance of a visual stimulus^12,13^. For example, it is logical to suppose that the chances of a successful catch in cricket slip-fielding may increase if the player has a fast VRT (resulting from rapid processing of visual information and subsequent generation of appropriate motor commands). Although the idea of a link between faster VRT and sporting excellence is appealing, the findings in high-level sports people are contentious. Although some studies report an inconclusive link between VRT and sporting expertise (e.g.^14–19^), there are a similar number that have reported a correlation between VRT and performance. For example, it has been reported that VRT are faster in elite than in sub-elite players^20,21^, as well as in elite players compared to non-players^22–30^. Faster VRT in elite sports players have also been linked to better performance on the field^31,32^.

What factors may contribute to such differing conclusions concerning the importance of VRT in elite sport? Putting aside differences in the samples studied and in the experimental protocols, one overlooked factor is gaze stability during VRT measurement. Blinks and saccadic eye movements both have the capacity to disrupt visual perception. Vision is temporarily occluded during blinks which typically last for around 200 ms^33^, and saccadic eye movements, whose duration ranges from 20 ms to more than 100 ms, depending on the amplitude of the movement^34^, result in rapid retinal image motion. Perceptually however, we are unaware of these frequent intrusions owing to suppression mechanisms: during suppression episodes, visual perception is briefly suspended^35,36^. In the case of both blink suppression and saccadic suppression, the suppression begins before the commencement of the blink or saccade and it ends after it^37,38^. It has been estimated that blink suppression lasts for around 200–250 ms^37^, while saccadic suppression lasts for a shorter period, estimated at 100–150 ms^38^. Accordingly, it follows that the ability to refrain from saccades and blinks (i.e., to control gaze stability) in the crucial period around stimulus onset could convey an advantage in VRT experiments because there will fewer periods when visual perception is suspended.

Johns et al.^39^ studied the influence of saccades and blinks on simple VRT in visually typical adults and found that VRT increased significantly, many by more than 200 ms, when a blink occurred from 75 ms before up to 150 ms after stimulus onset. A similar result was observed with saccades that started 75 to 150 ms after stimulus onset. It remains an open question, therefore, whether the reported faster VRT of elite athletes are a consequence of better gaze stability during the critical time of stimulus presentation during the VRT task. To that end, we adopt the protocol used by Johns et al.^39^ to study the effect of gaze stability on VRT in elite cricket and rugby league players compared to non-sporting controls. We measured VRT and recorded when and how many saccades and blinks took place relative to stimulus onset. We hypothesised that evidence of faster VRT in athletes may be related to better gaze stability, specifically fewer saccades and blinks at critical times relative to stimulus onset. We used an opportunity sample of participants comprising athletes from two very different sports and non-sporting controls, thus enabling us to investigate whether there was a sport-specific impact of gaze stability on VRT. If faster VRT in elites originates from better gaze stability, we expect to see similar patterns in the results for our rugby players and cricketers compared to the controls (i.e. non-sport-specific advantage). However, since the visual demands of cricket and rugby are very different (notably in relation to the requirements for gaze stability), it is possible that the pattern of results for the cricketers may differ relative to both the rugby players and the controls (i.e. a sport-specific advantage).

## Results

### Reaction time: group main effects

**Figure 1:**
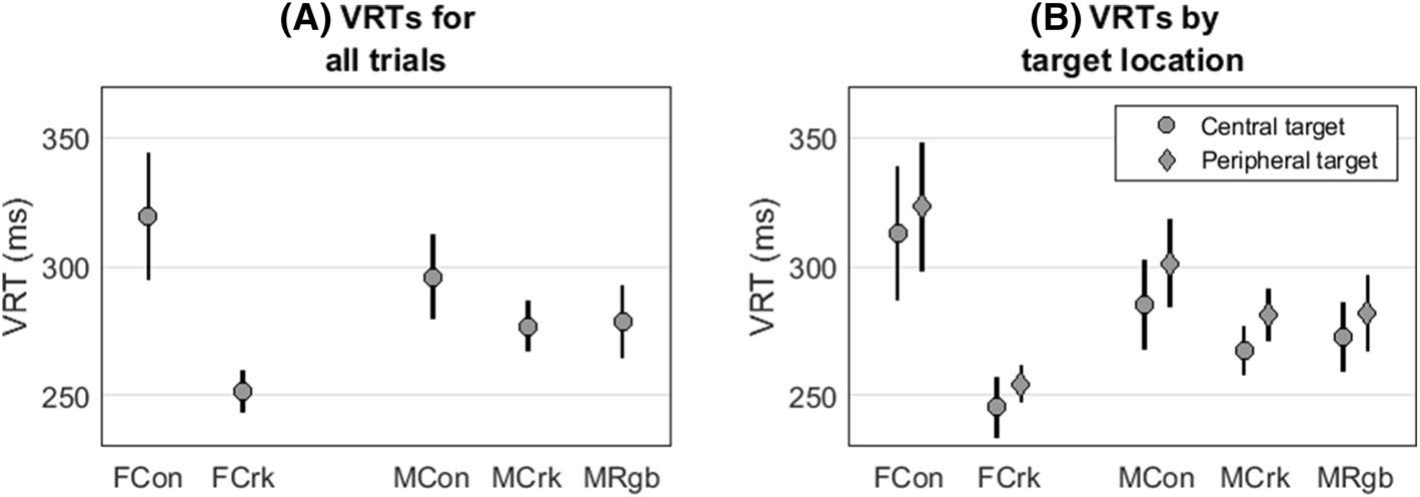
Mean (± 95% confidence interval) VRT following target appearance for female controls (FCon), female cricketers (FCrk), male controls (MCon), male cricketers (MCrk), and male rugby players (MRgb): (**A**) VRT for all trials; (**B**) VRT for each group split by central/peripheral target presentation.

VRT is defined here as the time between the onset of a visual stimulus (presented either centrally, or peripherally to the right or left of fixation in random order) and the instant when the participant pressed a button in response to the stimulus onset. VRT of female controls were on average 62.6 ms slower than those of female cricketers (311.9 versus 249.3, *p* < 0.001). VRT of male controls were 19.2 ms slower than those of male cricketers (296.0 versus 276.8, *p* = 0.035). Although the male controls’ VRT were on average 17.4 ms slower than those of the male rugby players, this difference was not statistically significant (296.0 versus 278.6, *p* = 0.078) (Fig. 1A).

### Reaction time: target location effects and variation across trials

Female participants’ VRT were 8.4 ms slower when the target was presented peripherally (7.5° left or right of center) compared to centrally (*p* < 0.001). For male participants, VRT were 10.8 ms (*p* < 0.001) slower for peripheral, compared with central targets (Fig. 1B). We found no evidence of practice effects: VRT did not vary significantly with trial number for female participants (+ 0.03 ms/trial, *p* = 0.468) or for male participants (+ 0.04 ms/trial, *p* = 0.121). Presentation location (central or peripheral) and trial number are accounted for in all of the following regression models but, since they are not the focus of this study, they will not be discussed further.

### Blink and saccade occurrence by group

**Figure 2:**
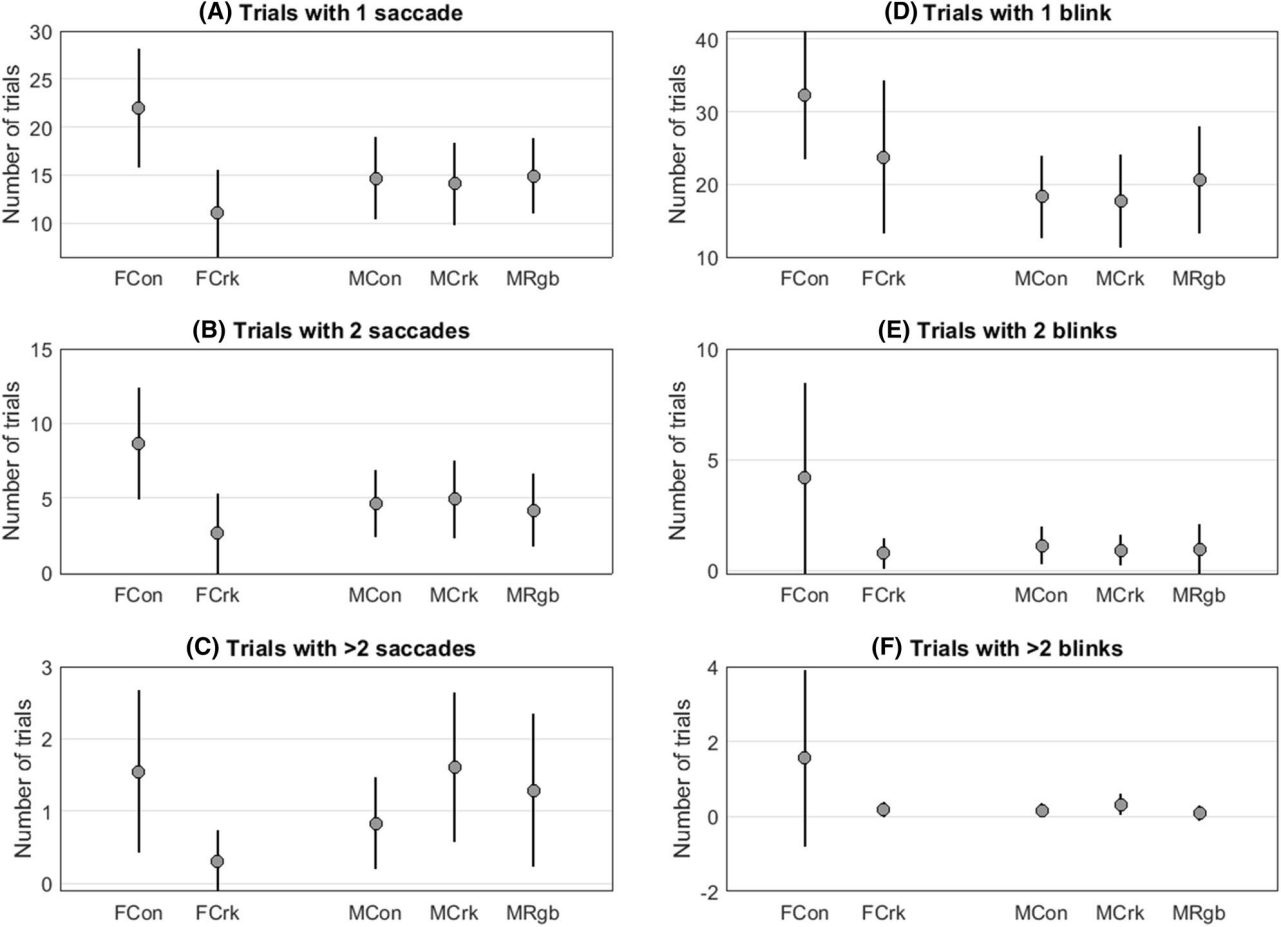
Group means for the number of trials containing blinks and saccades (lower values indicate better gaze stability). Error bars represent the 95% confidence intervals of group means. Female controls (FCon); female cricketers (FCrk); male controls (MCon); male cricketers (MCrk); male rugby players (MRgb).

We examined if the number of saccades and blinks differed between the elites and the controls (Fig. 2). Using a Poisson regression model, we found no difference in the number of saccades between the male athletes and male controls (*p* = 0.761). However, there was a significant difference in the females, with fewer saccades in the cricketers compared to the controls (*p* = 0.001). Similarly, using the same statistical approach, we found no significant difference in the number of blinks between male elites and male controls (*p* = 0.894) but significantly fewer blinks in females cricketers compared to female controls (*p* = 0.034). These results show that elite female cricketers had more stable gaze than the female controls.

**Figure 3:**
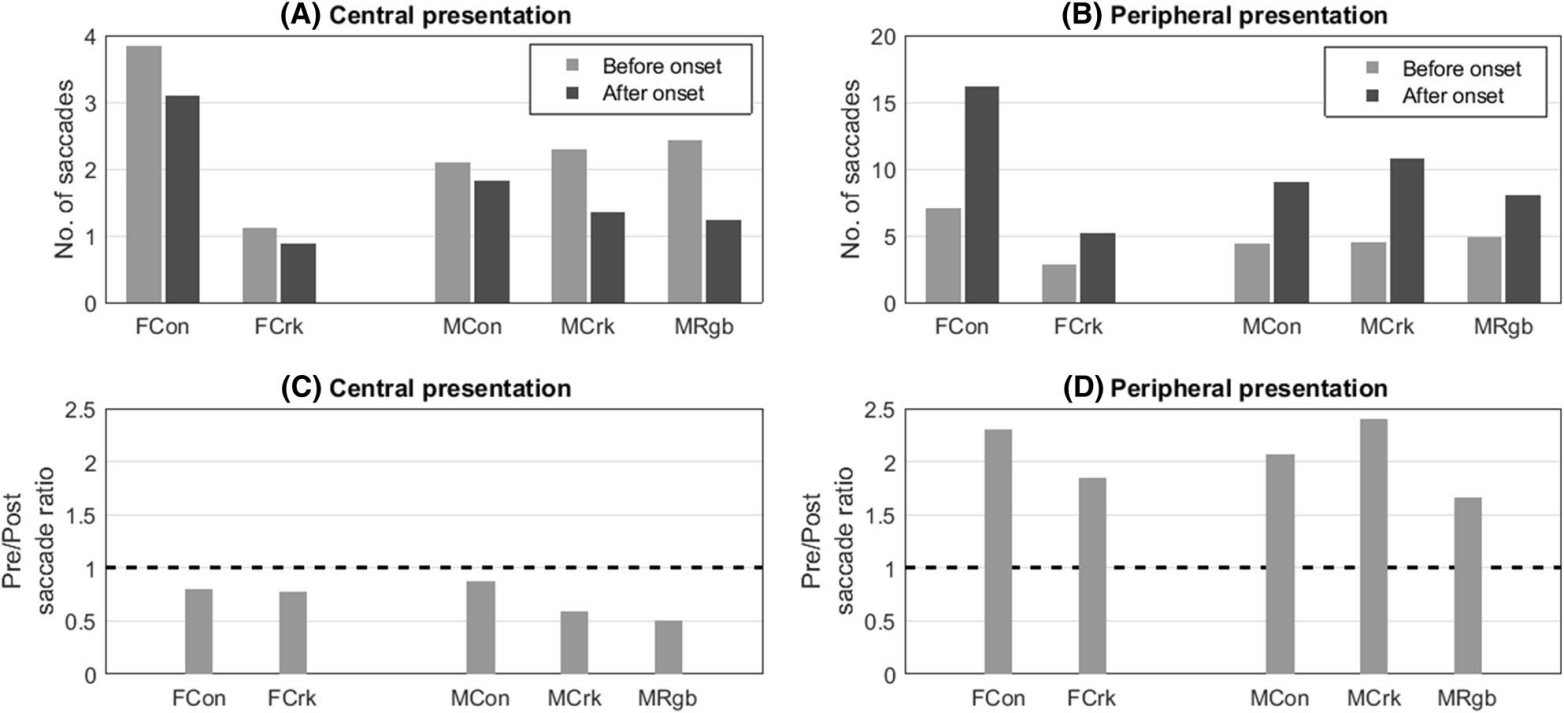
Mean number of saccades per group before and after target onset are plotted in panels (**A**,**B**), for central and peripheral locations, respectively. The ratios of saccades occurring pre and post onset for central and peripheral target presentations are shown in panels (**C**,**D**), respectively. Female controls (FCon); female cricketers (FCrk); male controls (MCon); male cricketers (MCrk); male rugby players (MRgb). Only saccades occurring from 225 ms before to 225 ms after presentation are included. Ratios are less than one where the number of saccades after target onset decreased compared to before target onset. Note that the mean values were calculated per participant from 30 trials and 60 trials for central and peripheral presentations, respectively (see “Methods” section).

To further compare the pattern of saccades in elites versus controls, we compared the number of saccades in the 225 ms prior to target onset with the number for the 225 ms period following target appearance. This comparison of saccades, before-onset versus after-onset, was made separately for central and peripheral target presentations (Fig. 3). For centrally presented targets there was a reduced number of saccades after onset compared to before onset for all groups (ratios of post-versus pre-saccade count range from 0.51 to 0.87). By contrast, there was an increase in the number of saccades after onset compared to before onset for peripherally presented targets in all groups (ratios of post-versus pre-saccades range from 1.66 to 2.4). Thus, although the female controls exhibited a larger overall number of saccades than the female cricketers (and indeed, than all other groups), this analysis shows that all groups displayed a similar tendency to saccade to the target location after the target had been presented in the periphery (Fig. 3). There were twice as many peripheral presentations as central presentations which explains why, for each group, the number of saccades before central target presentation is around half that before peripheral target presentation.

### Reaction time: impact of multiple blinks and saccades

Across all groups, 24.2% of trials contained a single blink, 1.7% contained two blinks and 0.5% contained 3 or more blinks. Similarly, 17.0% of trials contained one saccade, 5.6% contained two saccades and 1.3% contained three or more saccades. Therefore, we examined if VRT were affected differently by the number of saccades or blinks, regardless of their occurrence in the − 450 ms to + 225 ms recording window. To this end, we compared VRT in trials with no blinks or saccades to: (i) trials with a single blink or saccade; (ii) trials with two or more blinks or saccades.

Compared to trials with no blinks, VRT increased in male participants if a single blink occurred (by 5.5 ms, *p* = 0.002); the increase in females was similar but the effect failed to reach statistical significance (5.0 ms, *p* = 0.053). Again, relative to trials without blinks, the increases in VRT were more marked when there were two (males: 23.1 ms, *p* < 0.001; females 23.7 ms, *p* = 0.001) or more blinks (males: 38.3 ms, *p* = 0.013; females 26.3 ms, *p* = 0.024), although the proportion of trials with more than two blinks is small (0.5%) so there are large standard errors associated with the model coefficients. With the occurrence of a single saccade, VRT increased in both female (by 13.6 ms, *p* < 0.001) and male (by 19.6 ms, *p* < 0.001) participants compared to trials with no saccade. VRT increased further when there were two (males: 28.6 ms, *p* < 0.001; females 19.9 ms, *p* = 0.001) or more saccades (males: 43.0 ms, *p* < 0.001; females 26.8 ms, *p* = 0.042), although the proportion of trials with three or more saccades is small (1.3%), particularly in females, and the standard errors associated with the model coefficients are again large. All *p*-values for interaction terms were above 0.2 indicating that the effect upon VRT of different numbers of saccades and blinks was similar across groups.

### Reaction time: influence of saccade timing relative to target onset

**Figure 4:**
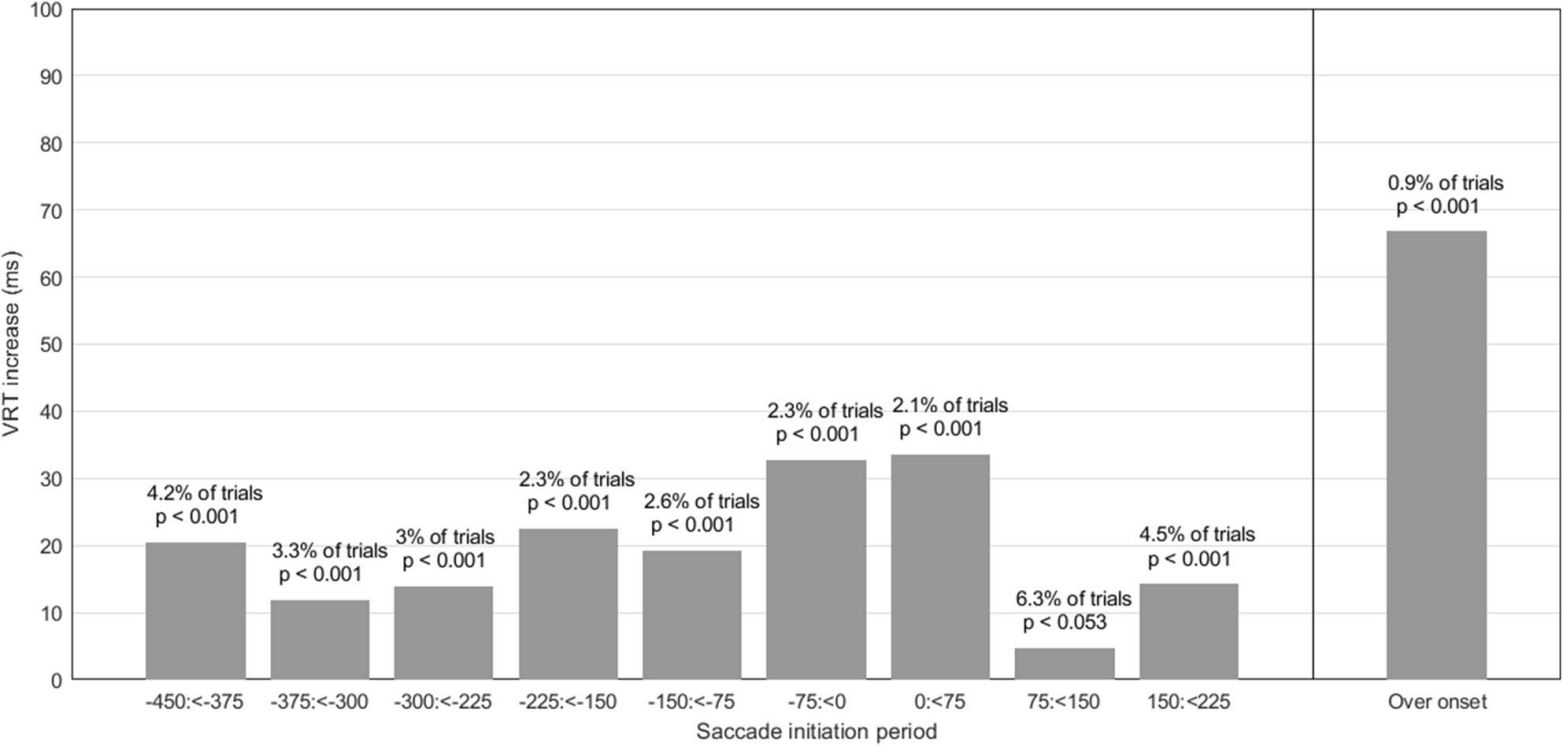
Effect on VRT of a saccade initiation at times relative to target appearance. On the x-axis, 0 refers to target onset. ‘Over onset’ refers to trials in which a saccade was in progress when the target appeared. For this reason, this bin contains trials with saccades that had various initiation periods relative to target onset. The increase in VRT (plotted on the y-axis) is the increase relative to trials on which there was no saccade in the period from 450 ms before target onset to 225 ms after onset.

We examined the extent to which the timing of saccade onset influenced VRT compared to trials in which no saccade occurred (Fig. 4). Saccades were grouped into 75 ms bins according to their initiation relative to target onset. These timing bins were then treated as categorical variables in a regression analysis.

Figure 4 shows the detrimental influence of a saccade on VRT was greatest (+ 66.8 ms) when the saccade overlapped target onset (*p* < 0.001). When a saccade began within 75 ms of target onset, VRT were markedly slower (by 32.8 ms for saccades in the period from − 75 ms to < 0, *p* < 0.001; by 33.6 ms for saccades initiated in the period from 0 to +  < 75 ms, *p* < 0.001) (Fig. 4). However, although saccades taking place during or very near to the target onset clearly lead to a marked increase in VRT, only a small proportion of trials had saccades at these crucial moments. Overall, only 2.3%, 0.9% and 2.1% of trials had saccades up to 75 ms before target onset, overlapping target onset or up to 75 ms after target onset. Having established earlier that the effect of presence of a saccade had the same effect across groups, we had no theoretical rationale to search for group-by-timing of saccades interaction terms.

### Reaction time: impact of blinks and saccades

**Figure 5:**
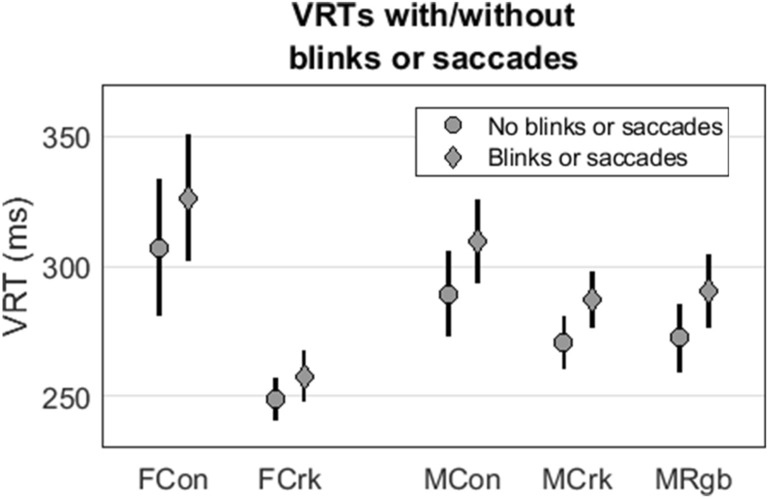
Mean (± 95% confidence interval) VRT following target appearance for female controls (FCon), female cricketers (FCrk), male controls (MCon), male cricketers (MCrk), and male rugby players (MRgb). VRT for each group are split by presence/absence of saccade(s) or blink(s). ‘Blinks or saccades’ (diamonds) refers to trials on which there was a blink or saccade in the period − 450 ms to + 225 ms relative to target onset. No blinks or saccades (circles) are trials on which there was no blink or saccade in this period.

We explored how the presence of a saccade or blink impacted on VRT. Group VRT split by presence/absence of saccades and blinks are shown in Fig. 5. We defined a saccade or blink as being present if it was initiated any time between 450 ms before, or 225 ms after, target onset. For female participants, the presence of a saccade in this period increased VRT by an average of 16.0 ms (*p* < 0.001) compared to trials in which no saccade had taken place in this interval. A blink in that period had a smaller effect, raising VRT by an average of 5.9 ms (*p* < 0.001). When the regression model was re-run using trials in which no blinks or saccades had taken place, VRT of female controls (304.3 ms) remained 64.1 ms slower than those of female cricketers (240.2 ms, *p* < 0.001).

For male participants, the presence of a saccade increased VRT by an average of 22.5 ms (*p* < 0.001) whereas a blink raised RTs by an average of 6.8 ms (*p* < 0.001). When the regression model was re-run using trials in which no blinks or saccades had taken place, VRT in controls (280.1 ms) remained, on average, slower than those of cricketers (19.2 ms, *p* = 0.025) and slower than those of the rugby players (17.5 ms, *p* = 0.059). In short, the group comparisons were virtually unchanged. Thus, we did not find evidence to support the hypothesis that better gaze stability explains faster VRT in elite sports players compared to controls, or that gaze stability can account for differences in VRT between sports with different visual demands.

## Discussion

We investigated whether gaze stability, as assessed by the incidence of saccades and blinks, influences VRT of elite athletes compared to non-sporting controls. Samples of international-level female cricketers, national-level male cricketers, professional-level male rugby players and female and male non-sporting controls responded to the appearance of a visual target presented either centrally or peripherally (providing the VRT measure) whist their saccades and blinks were monitored (providing a measure of gaze stability). Our results show that (1) cricketers, but not rugby players, have faster VRT than controls; (2) when they occur, blinks and, in particular, saccades are associated with slower VRT regardless of the level of sporting ability; (3) elite female cricketers had steadier gaze (fewer trials with saccades and blinks) in comparison to female controls, but gaze stability did not differ between the male elites and controls; (4) while gaze stability does affect VRT and gaze stability may differ between elite and control groups, the stability of gaze was not a factor that explains the difference between elite and control groups in VRT. When we accounted for the presence of blinks and saccades, our group comparisons of VRT were virtually unchanged. Thus we conclude that better gaze stability does not explain faster VRT in elite sports players compared to controls.

There is mixed evidence in favour of (e.g.^20,21,23–30^) and against (e.g.^14–19,22^) faster VRT in elite athletes. The evidence in the current study is similarly mixed. All our elite groups had faster mean VRT than the control groups but the difference between groups was not consistently significant. Male and female cricketers’ VRT were significantly shorter than gender-matched controls but the VRT of the rugby players did not differ significantly from gender-matched control subjects (Fig. 1). We should emphasize that absolute differences in VRT between elites and controls, and differences in VRT between the genders, are of secondary interest in this study as we were primarily concerned with whether any differences in VRT between elite and controls can be explained by taking account of gaze steadiness as measured by the number and timing of blinks and saccades.

In agreement with Johns et al.^39^, we found that participants often made a saccade and/or blink during the − 450 ms to + 225 ms period around target onset (Fig. 3). Also, we found that a blink or saccade at any point in this period led to slower VRT and that more blinks and saccades led to greater increases in VRT. The impact of saccades on VRT was particularly dramatic when they occurred close to the instant of target onset, and the impact was greatest when they overlapped target onset (Fig. 4). Though the occurrence of blinks and saccades was similar in all three male groups, the female controls exhibited significantly more blinks (*p* = 0.001) and saccades (*p* = 0.034) than the female cricketers (Fig. 2). Despite this, we found no evidence that the occurrence of blinks and saccades accounted for differences in VRT between elites and controls in either the male or female groups since group differences were virtually unchanged when we took account of trials in which blinks and saccades had taken place. Importantly, participants in the present study were asked to fixate on a cross at the centre of the screen and to maintain their gaze on that location throughout the trial. The saccades and blinks which were initiated prior to, and coincident with, the onset of the target represented a failure to hold the eyes open and steady during the information gathering phase of the trial. Saccades initiated in the period 150 to 225 ms after target onset are likely to be related to an inability to inhibit a pro-saccade, which takes place following the appearance of a target in the visual scene^40^.

A number of previous studies have shown that VRT’s increase if the target is presented after the saccade has been initiated^41,42^. Those studies attempted to separate the perceptual and motor components of the VRT task and to identify how saccades may influence each component. In Baedeker and Wolf^41^, visually-evoked potentials (VEPs) were measured following stimulus onset, both in the presence and absence of deliberately-executed saccades. The authors found VEP latencies in the saccade and no-saccade conditions to be almost identical. On this basis, the authors concluded that the increase in VRT which occurs in the aftermath of a saccade was not due to slower perceptual processing following the onset of the stimulus on the screen, but was instead due to interference in the execution of the motor task. In other words, initiating the saccade interfered with the ability to execute the manual response (i.e. the button press) in the VRT task. Interestingly the size of this interference effect has been reported to be much smaller in volleyball players than in non-athletes^43^. The issue of whether faster VRT’s arise because of earlier processing of visual signals or from accelerated motor processes continues to be the subject of considerable research interest. A number of recent studies have featured VRT measures in participants in whom electrophysiological data have simultaneously been gathered^28,30^. For example, in a study of elite badminton players versus non-athletic controls^28^, faster VRT were found amongst the elites, and the origin of this superior performance was primarily associated with faster visual perception, with differences in motor-related processing time playing a comparatively minor role. In subsequent studies by the same group^13,30^, it was again concluded that VRT are predicted by the speed of visual processing in elite badminton players.

Regardless of whether the finding of faster VRT arise from faster perceptual or quicker motor-related processing, there is considerable doubt about the significance of faster VRT’s for elite sporting performance, even in time-critical sporting scenarios. While some authors continue to make the claim that faster VRT’s are associated with elite performance, and that explicit training may improve VRT and thus lead to better sporting performance (e.g.^13^), an alternative and widely held view is that the elite advantage is based on their ‘perceptual-cognitive expertise’^1–11^. The latter is underpinned in part by knowledge about precisely where and when to look in order to gather the key information that enables the elite-level player to anticipate the events that are about to unfold and thus to plan and execute the task at hand (see “Introduction” section). In demanding sporting scenarios, it becomes increasingly important that gaze is ‘precisely controlled in space and timed relative to specific phases of the motor skill’^44^. For example, exhibiting more fixations of longer-durations on task-relevant areas is associated with better performance in goal-keeping^6^, golf putting^1^, and many other sporting scenarios^45^. Our results indicate that training to increase gaze stability is unlikely to lead to quickening of VRT, though as indicated above, there may be advantages in training patterns of gaze control to enhance information gathering/processing and the planning of motor responses^46^.

## Methods

### Participants

We recruited our participants for this study using an opportunity sampling approach. Our sample consisted of five groups: an elite cricket group (female); a near-elite cricket group (male); an elite rugby group (male); a male control group; and a female control group. Elite cricketers were members of England’s national women’s cricket team (n = 16, 25.0 ± 2.9 years). Our male cricketers were members of the Leeds/Bradford Marylebone Cricket Club University squad (all male, n = 28, 21.0 ± 1.5 years) which comprises the best young players drawn from universities in Yorkshire. This team plays fixtures against English county-level sides. We had access to male rugby players, though unfortunately not to an elite, female rugby sample. Elite rugby players (n = 21, 23.0 ± 4.0 years) were members of a professional, ‘Super-League’, Rugby-League squad. We included elites from more than one sport for the reasons outlined in the introduction. Both control groups were students at the University of Bradford who had never played ball sports at a competitive level and who did not routinely play ball-sports (male controls: n = 30, 23.0 ± 7.0 years; female controls: n = 20, 22.0 ± 4.0). Protocols were approved by the Committee for Ethics in Research at the University of Bradford and were in accord with the tenets of the Declaration of Helsinki. Participants gave written informed consent and reported normal or corrected-to-normal vision and no known neurological or sensorimotor deficits.

### Equipment and procedure

Participants sat in a darkened room at a chin-rest, 61 cm from a Sony (Sony Corporation, Tokyo, Japan) Trinitron CRT monitor (19.5″, 100 Hz refresh rate, 1,024 × 768 pixel resolution) connected to a HP Z220 Workstation (Hewlett-Packard, Palo-Alto, CA). The software controlling the presentation was written in SR Research Experiment Builder 1.10.1421 (SR Research Ltd., Ontario, Canada).

Participants’ eyes were level with the centre of the screen. The procedure was self-paced in that the participant was asked to press the space bar on a keyboard to begin each trial, at which point a white fixation cross appeared in the centre of the screen. Participants were asked to maintain fixation on the cross, and to press the keyboard space bar (1000 Hz., Razer RZ03-0018, Razer Inc., San Francisco, CA) as soon as the white target appeared against a grey background. A 20-in Sony Trinitron GDM-F520 CRT monitor (Sony Corp., Tokyo, Japan), with a refresh rate of 120 Hz, was used to display the target and background. The target was circular with a diameter of 0.8° and could appear with a probability of 0.33 centrally, or 7.5° to the left or 7.5° to the right of fixation. The viewing distance was 61 cms. The target remained on the screen until the participant responded. Participants were told that they would hear an error tone if they pressed the space bar before the target appeared, in which case that trial was rejected (see below for proportion of trials rejected).

Eye movements were recorded with an Eyelink 1000 (SR Research Ltd., Ottawa, Ontario, Canada), recording monocularly from participants’ dominant eye, at 250 Hz. Eye dominance was ascertained using a modified version of the hole-in-the-card test. After a 9-point calibration and validation for the eye-tracker, participants completed 9 practice trials: a random arrangement of 3 trials for each target location. Participants were given the opportunity to ask any questions about the procedures to be followed during the training phase. The main experiment consisted of 90 trials, separated into 3 blocks of thirty trials, with each block having a random arrangement of 10 left-sided, 10 central and 10 right-sided presentations. At the end of each block a screen appeared, offering participants the chance to take a rest. No feedback was given, except for a tone if the response preceded the target appearance.

Saccades were detected using the standard SR definition of the saccade, which is based on velocity (eye movement of > 30°/s) and acceleration (acceleration of > 8,000°/s^2^). A saccade was counted if any part of it occurred in the interval of 450 ms prior to target appearance to 225 ms after it. Like saccades, blinks were also counted over the time interval − 450 ms to + 225 ms relative to target onset. The impact upon VRT of the timing of saccade initiation relative to target onset was studied for saccades. We were not able to perform the equivalent analysis for blinks, because although we were aware of the number of blinks that took place that in the − 450 ms to + 225 ms time window, the data were not coded according to when blinks had taken place relative to target onset.

### Statistical analysis

Data were analyzed using random-effects modelling with maximum-likelihood estimation in STATA (version 13, StataCorp LP, College Station, TX), conducted separately for males and females because of the well-established gender differences in simple RT^47^. Trial number was treated as a co-variate, whereas skill-level and presentation location (central or peripheral) were treated as categorical (fixed effects) variables. This analysis allows for the likelihood that the effects will vary between participants.

Separate models were run where blinks and saccades were treated as a binary factor (present/absent), and where saccades were treated as a categorical variable reflecting the timing of each saccade relative to target onset. We also examined how VRT were affected by the presence of more than one blink and/or saccade.

### Data exclusion

Plots are generated from participant means following data exclusion. Trials in which VRT (to stimulus onset) were < 50 ms or > 750 ms were excluded from the analysis because such VRT were deemed implausible and/or erroneous. The percentage of excluded trials was low; the group with the highest proportion of excluded trials was the male controls and only 1.2% of trials from this group were excluded. The remaining data (available as a supplementary file) were the data analysed.

## Supplementary information

Supplementary Information
